# Mitochondrial DNA Fitness Depends on Nuclear Genetic Background in *Drosophila*

**DOI:** 10.1534/g3.119.400067

**Published:** 2019-02-11

**Authors:** Jim A. Mossman, Jennifer Y. Ge, Freddy Navarro, David M. Rand

**Affiliations:** * Department of Ecology and Evolutionary Biology, 80 Waterman Street, Box G, Brown University, Providence, Rhode Island 02912; † Department of Medical Oncology; ‡ Department of Biostatistics and Computational Biology, Dana-Farber Cancer Institute, 450 Brookline Ave, Boston, MA 02215; § Harvard-MIT Division of Health Sciences and Technology, Harvard Medical School, 25 Shattuck St, Boston, MA 02115

**Keywords:** mitochondrial DNA, epistasis, fitness, *Drosophila*, introgression, haplotype, reperturbation cages

## Abstract

Mitochondrial DNA (mtDNA) has been one of the most extensively studied molecules in ecological, evolutionary and clinical genetics. In its early application in evolutionary genetics, mtDNA was assumed to be a selectively neutral marker conferring negligible fitness consequences for its host. However, this dogma has been overturned in recent years due to now extensive evidence for non-neutral evolutionary dynamics. Since mtDNA proteins physically interact with nuclear proteins to provide the mitochondrial machinery for aerobic ATP production, among other cell functions, co-variation of the respective genes is predicted to affect organismal fitness. To test this hypothesis we used an mtDNA-nuclear DNA introgression model in *Drosophila melanogaster* to test the fitness of genotypes in perturbation-reperturbation population cages and in a non-competitive assay for female fecundity. Genotypes consisted of both conspecific and heterospecific mtDNA-nDNA constructs, with either *D. melanogaster* or *D. simulans* mtDNAs on two alternative *D. melanogaster* nuclear backgrounds, to investigate mitonuclear genetic interactions (G x G effects). We found considerable variation between nuclear genetic backgrounds on the selection of mtDNA haplotypes. In addition, there was variation in the selection on mtDNAs pre- and post- reperturbation, demonstrating overall poor repeatability of selection. There was a strong influence of nuclear background on non-competitive fecundity across all the mtDNA species types. In only one of the four cage types did we see a significant fecundity effect between genotypes that could help explain the respective change in genotype frequency over generational time. We discuss these results in the context of G x G interactions and the possible influence of stochastic environments on mtDNA-nDNA selection.

During the last half century, studies on mitochondrial biology have revolutionized the fields of metabolism, aging, evolutionary processes and organismal fitness. Seventy years ago it was hypothesized that mitochondria exclusively synthesize adenosine triphosphate (ATP) via oxidative energy metabolism (Ephrussi *et al.* 1949; Kennedy and Lehninger 1949). Now, the precise roles of mitochondria in the cell are understood to be far more comprehensive, involving many biosynthetic and degradative reactions including metabolism of amino acids, lipids and iron, and programmed cell death (apoptosis) (Tzagoloff 1982; Pfanner *et al.* 2004). More recently, the role of mitochondrial ‘performance’ and its associated genetic variation has been suggested to underpin a grossly underestimated number of human diseases and fitness-related phenotypes (Schon *et al.* 2012).

Mitochondrial biogenesis is jointly encoded by both mitochondrial and nuclear genomes. The nuclear DNA encodes ∼1200 mitochondrial genes whereas the mitochondrial DNA (mtDNA) encodes 37 (13 protein coding, 22 transfer RNAs and 2 ribosomal RNAs) (Smeitink *et al.* 2001). This co-evolved mito-nuclear gene complex provides an interesting target for studies of selection for two main reasons. First, the genome inheritance patterns differ; mtDNAs are maternally inherited, whereas nuclear DNAs (nDNA) are biparentally inherited. Second, mutations in nDNA and mtDNA genes that encode for proteins of the oxidative phosphorylation (OXPHOS) pathway are associated with deleterious phenotypes, including mitochondrial diseases (Wallace 1999; Smeitink *et al.* 2001), organismal longevity (Rand *et al.* 2006; Camus *et al.* 2012; Clancy 2008; De Benedictis *et al.* 1999), and measures of overall organismal fitness (Ballard and James 2004; Ballard and Rand 2005; Gerber *et al.* 2001).

Mutations in mtDNA are predicted to have phenotypic consequences on the organism and to date there is good evidence that both point mutations (SNPs) and large scale deletions in mtDNA have been shown to affect the organismal phenotype (Schon *et al.* 2012; Taylor and Turnbull 2005). Interestingly, mtDNA mutations principally manifest in tissue types with a high metabolic demand, *i.e.*, where ATP production is normally highest (Wallace 2005, 1999). It is therefore not surprising that an organelle that provides ∼90% of ATP to the cell may be sensitive to mutation, and that those mutations confer phenotypic consequences. However, the precise roles of mutations in either genome on the whole organism have been historically difficult to disentangle (Rand *et al.* 2004).

Given that mtDNA and nDNA have co-evolved over evolutionary time, it is suggested that disruption of co-evolved mito-nuclear gene complexes will be deleterious to organisms, when non-coevolved combinations are compared to coevolved mito-nuclear combinations (Hutter and Rand 1995; Rand *et al.* 2004). Moreover, the degree of disruption of the co-evolved state is expected to correlate with phenotypic effects although explicit tests of this hypothesis are unresolved across species (but see Clark and Lyckegaard 1988; Montooth *et al.* 2010; Camus *et al.* 2012). We would predict that higher numbers of mtDNA mutations from the co-evolved complex would have a greater phenotypic effect than lower numbers and that the magnitude of the phenotypic response to genetic disruption would be correlated with the degree of genetic disruption.

Previous mtDNA-nDNA fitness studies have largely concentrated on either natural genetic variation or within-species introgression in *Drosophila* (Clark and Lyckegaard 1988; Macrae and Anderson 1988; Fos *et al.* 1990; Garcia-Martinez *et al.* 1998; Rand 2001; James and Ballard 2003; Ballard and James 2004; Dowling *et al.* 2008). Within-species mtDNA-nDNA introgression experiments have demonstrated various effects on fitness, including: (i) haplotype frequency changes in population cages in *D. pseudoobscura* (Macrae and Anderson 1988), *D. melanogaster* (Kilpatrick and Rand 1995), and *D. simulans* (Ballard and James 2004); (ii) changes in nuclear gene expression in *D. melanogaster* (Innocenti *et al.* 2011); (iii) aging phenotypes in *D. melanogaster* (Camus *et al.* 2012); (iv) sperm competitiveness in *D. melanogaster* (Yee *et al.* 2013), but see Friberg and Dowling 2008; (v) development time, survival probability, and organismal activity in *D. simulans* (James and Ballard 2003); (vi) development time in *D. melanogaster* (Mossman *et al.* 2016a); and (vii) female fitness in *D. melanogaster* (Dowling *et al.* 2007).

Investigating genetic components of fitness in natural populations is somewhat constrained by the lack of precise control over genotypic variation and the extent of genetic polymorphism and sequence divergence in study populations (Lewontin 1974). *Drosophila* genetics has proven to be a powerful tool to avoid these issues and to precisely manipulate genetic variation in a phylogenetic framework. For example, heterospecific mtDNA-nDNA introgression can be achieved due to the generation of reproductively viable hybrids between various species pairs. One example of this hybrid viability is between *D. melanogaster* and *D. simulans*. *Drosophila simulans* is a sister species to *D. melanogaster* and the separate species do not freely interbreed (Sturtevant 1920). However, the offspring arising from a cross between a female *D. simulans* (*C167.4* strain) and a *D. melanogaster In(1)AB* male are reproductively viable (Davis *et al.* 1996). The resulting inter-specific hybrids, while not present in nature, provide an opportunity to tease apart: (i) nDNA effects; (ii) mtDNA effects; and (iii) nDNA x mtDNA interactions (epistases), in populations of flies that probably demonstrate greater mtDNA-nDNA sequence divergence (and phenotypic variation) than flies found in natural populations.

There is good reason to introgress genomes between closely-related species. One of the pioneering studies of *Drosophila* cytoplasmic-nuclear introgression clearly demonstrated that the greatest effects on 2^nd^ chromosome and cytoplasm segregation patterns occurred when genetic material was exchanged between geographically isolated populations, with negligible effects observed when genetic material was exchanged within a population (Clark and Lyckegaard 1988). Introgressing genomes between-species essentially mimics and exaggerates this genetic polymorphism and the potential to detect mtDNA-nDNA interactions. Heterospecific mtDNA-nDNA introgressions in *Drosophila* have previously revealed mtDNA-nDNA epistases for fitness, including: (i) haplotype frequency changes in population cages of *D. persimilis*- *D. pseudoobscura* introgressions (Hutter and Rand 1995); and (ii) development time, bristle size and fecundity (Meiklejohn *et al.* 2013; Montooth *et al.* 2010; Mossman *et al.* 2016a), and aging phenotypes (Rand *et al.* 2006) in *D. simulans* - *D. melanogaster* mtDNA-nDNA introgressions. However, no study to date has investigated the relative fitness of mtDNA haplotypes in *D. simulans* – *D. melanogaster* introgressed fruit flies in a competitive context.

Comparisons of mtDNA coding sequence variation reveal an approximately fivefold difference in amino acid divergence within- and between- species in the *D. melanogaster*- *D. simulans* clade. For example, there are 18 amino acid substitutions between mtDNA haplotypes within *D. melanogaster*, and up to 45 amino acid substitutions between mtDNA haplotypes within *D. simulans*. Furthermore, the between-species comparison demonstrates up to 103 amino acid substitutions between *D. melanogaster* and *D. simulans* ((Ballard 2000); and see (Montooth *et al.* 2010) for all haplotype pairwise substitutions). These pairwise comparisons reveal two key features of this heterospecific introgression model that are important to formulate predictions for the current investigation. First, the degree of amino acid divergence within *D. simulans* is at least twice that of *D. melanogaster*. If we assume a greater level of mtDNA polymorphism within a species can potentially confer greater fitness differences than lower levels, then we would predict *a priori* that there would be greater divergence in phenotypes in the *D. simulans* clade than in the *D. melanogaster* clade. Second, the amino acid divergence within either species’ haplotypes is less than half the potential number of differences between species. A second prediction can thus be formulated that if tested, we would expect more phenotypic divergence between clades than within a clade. In view of the fact that in the *D. simulans – D. melanogaster* introgression model the mtDNA haplotypes are all resident on *D. melanogaster* nuclear backgrounds, we can further predict that the non-coevolved mtDNA haplotypes (in the *D. simulans* clade) will demonstrate deleterious phenotypes if compared to *D. melanogaster* mtDNA haplotypes. Is there any evidence, though, for differential fitness in the *D. simulans* haplotypes when on their native *D. simulans* nuclear background?

Cytoplasmic (including mtDNA) micro-injection experiments performed within *D. simulans* nDNA flies have previously shown differential fitness of *D. simulans* mtDNA haplotypes, judged as levels of heteroplasmy; a measure of mtDNA competitive exclusion (De Stordeur 1997). Assessed mtDNAs were from the monophyletic *si*I, *si*II and *si*III haplotypes. Overall, the rank fitness of the haplotypes corresponded to *si*II>*si*III>*si*I (De Stordeur 1997). In a separate examination using the same mtDNA haplotypes, development times (egg-to-puparium, and egg-to-eclosion) were shown to be longest in *si*II and *si*III flies, and shortest in *si*I flies (James and Ballard 2003). A follow-up study showed repeatable haplotype frequency changes in perturbation-reperturbation experiments (Ballard and James 2004), corresponding to a rank fitness of *si*II>*si*III>*si*I; the same as in De Stordeur (1997). There therefore appears to be functional variation between these haplotypes on a *D. simulans* nuclear background.

The observed fitness effects of alternative mtDNA haplotypes on a *D. simulans* nDNA background allows us to now ask the question: are the effects associated with mtDNA variation alone, or are they mediated by nuclear genetic variation? Previous studies have explicitly conducted experiments on a fixed nuclear background to eliminate this potential source of fitness variation. The heterospecific mito-nuclear introgression model provides a powerful experimental tool to test for repeatable main effects of mtDNA variation, yet crucially permits a simultaneous test of whether the rank order of mtDNA fitness changes on an alternative nuclear background (to test whether mtDNA selection is repeatable or not). Altered fitness on different nDNA backgrounds would provide good evidence for a mtDNA-nDNA epistasis for fitness.

Here, we tested whether there is any genetic variance for fitness of *D. melanogaster* and *D. simulans* mtDNA haplotypes when on *D. melanogaster* nDNA backgrounds. Our investigation was divided into separate species comparisons, so *D. simulans* mtDNA haplotypes were competed against each other and *D. melanogaster* mtDNA haplotypes were competed against each other. We did not compete *D. melanogaster* mtDNAs against *D. simulans* mtDNAs. Specifically, we explored the effect of genome introgressions in population perturbation- reperturbation cages. Perturbation cages allow the monitoring of haplotype frequency over discrete, non-overlapping generations of breeding to determine gross estimates of competitiveness, or fitness, between different mtDNA haplotypes (Arnason and Lewontin 1991; Hutter and Rand 1995). By reperturbing the population after a given number of generations it is possible to re-set the populations back to the original equal haplotype frequencies and observe the repeatability of haplotype selection coefficients, pre- and post- perturbation. Perturbation-reperturbation also allows subtle changes in nuclear genetic effects to be detected pre- and post- perturbation and allows delineation of selection and drift processes. Each population cage contained only one nuclear genetic background. In addition to the population cages, we also tested the fitness (fecundity) of introgressed genotypes in a non-competitive context to observe whether the fitness differences observed in population cages could be explained by the numbers of offspring produced by females of known mtDNA-nDNA introgressions.

## Materials and Methods

### Fly stocks

MtDNA-nDNA introgression flies, in which alternative nuclear DNA backgrounds have been precisely placed on different mtDNA haplotypes using balancer chromosomes, were used for this experiment. We used balancer chromosomes to effectively introgress genomes instead of reciprocal back-crossing because the latter procedure may retain nuclear variants from maternal parents during the back-crossing process (see Montooth *et al.* 2010). In brief, two nuclear backgrounds were used corresponding to the inbred laboratory strain Oregon R (*OreR*) and a wild-caught strain from Austria (*AutW132*, hereafter named *Aut*), described in Montooth *et al.* (2010). Both nuclear types were introgressed with either: (i) three *D. simulans* mtDNA haplotypes separately (*mau*12, *si*I, and *sm*21) corresponding to the monophyletic *D. simulans si*III, *si*I, and *si*II haplotypes, respectively, or (ii) two *D. melanogaster* mtDNA haplotypes separately (Zimbabwe 53 (*Zm53*) and Oregon R (*OreR*)). These generated flies are viable and have been previously used to research the effects of mito-nuclear epistases on fitness (Montooth *et al.* 2010). Flies used in this experiment had previously been tetracycline-cleared to eliminate confounding effects of *Wolbachia* infection.

### Perturbation reperturbation cages

To monitor the change in mitochondrial haplotype frequency over time, perturbation reperturbation cages were constructed to house ∼700 flies whose starting haplotype frequency was equal between haplotypes, following a similar procedure to Ballard and James (Ballard and James 2004). In total, the 16 experimental cages comprised of 8 cages with *OreR* nuclear genomes and 8 cages with *Aut* nuclear genomes. Each nuclear genome cage type was initiated with either: (a) each of three *D. simulans* mitochondrial haplotypes (*si*I, *mau*12 and *sm*21: starting frequency 33% each), or (b) each of two *D. melanogaster* mitochondrial haplotypes (*Zm*53 and *OreR*: starting frequency 50% each). *Mau*12 is a haplotype from *D. mauritiana* that differs by 1-2bp when compared to the *D. simulans si*III haplotype, and is therefore phylogenetically equivalent to *si*III. Each mtDNA-nDNA cage trial had four replicates per treatment. A population size ∼700 was targeted throughout to minimize the effects of fluctuating effective population size or genetic drift on mtDNA haplotype frequencies. Population sizes in each cage are reported in the Supplementary Materials Table S2 and Figure S4. Cages were maintained at 25° on a [12hr: 12hr] light: dark cycle. Prior to the experiment, flies from each haplotype were maintained at a controlled density for two generations.

To test the repeatability of haplotype frequency change over time, the experiment was divided into two sections; pre-perturbation and post-perturbation. The experimental food was a standard 2% yeast diet with no additional sprinkled yeast (11% sugar, 2% autolyzed yeast, 5.2% cornmeal, agar 0.79% w/v in water and 0.2% tegosept -methyl 4-hydroxybenzoate, from Sigma (St. Louis, MO, USA). Food (approximately 100ml) was added to a deep-sided petri dish (Fisher Scientific, Pittsburg, PA, USA). In the first generation, cages were seeded with presumably- mated five day old females (233 per genotype in *D. simulans* cages (699 total) and 350 per genotype in *D. melanogaster* cages (700 total)). Females were separated from males at ∼4 days old and allowed to recover for one day prior to addition into population cages (when no CO_2_ knock-down was used). These seeding females were allowed to lay eggs for 5 days. After 5 days of egg laying flies were removed and kept for genotyping (generation 0 estimate). The eggs in the petri dish were allowed to hatch and the adults eclose. After 14 days post egg laying adults were knocked-down using CO_2_ and transferred to a fresh population cage with a new petri dish and food. The freshly-transferred adults were allowed to lay eggs for 5 days and the process was repeated for subsequent generations. The pre-perturbation stage lasted 9 generations and in the 10^th^ generation 48 presumably mated females were removed from each cage and each female was isolated into an individual vial to establish an isofemale line. The isofemales were allowed to lay eggs for 5 days and were then removed for genotyping. When the haplotypes of the isofemales from each cage were known (see genotyping protocol, below), all isofemale offspring of the same haplotype were pooled and mixed, and then the reperturbation experiment was conducted. The 11^th^ generation (generation 0 post-reperturbation) was founded with equal numbers of individuals from the respective haplotypes of that cage (as in the initial set-up, above). The post-reperturbation episode lasted 13 generations and flies were sampled at generations 0, 6, and 13.

### Non-competitive fecundity assay

To measure the fitness of different mtDNA haplotypes in a non-competitive environment, we first isolated 48 presumably mated isofemales from each of 16 cages at generation 10 pre-perturbation (see above) and allowed them to lay eggs, singly in single vials, for 5 days on standard 2% yeast-sugar-cornmeal medium with no added surface yeast (see above). After 5 days of egg laying, the isofemales were genotyped (see below) to determine their mtDNA haplotype. All flies from the same cage type have a known nuclear background and it was not necessary to genotype at nuclear loci. We counted the number of total eclosed offspring from each vial (16 cages × 48 vials = 768 isofemale vials that could be scored for a known haplotype). The number of offspring per vial was scored blind to the mtDNA haplotype identity. Following counting, the eclosed offspring of known haplotype were pooled to re-start the post-perturbation phase of the experiment (see above). The numbers of individuals of each haplotype scored for fecundity therefore represents a highly similar haplotype frequency of the generation nine populations.

### DNA extraction

For pre-perturbation experiments flies were sampled at the first two generations (generations 0 and 1) and subsequently every 2^nd^ generation (generation 3, 5, 7, 9). The post perturbation sampling frequency was the first (introduced) generation, then at generations six and thirteen. In total, for *D. simulans* haplotype cages, 92 flies were sampled per cage following the methods outlined in (Kilpatrick and Rand 1995), allowing up to three positive controls (corresponding to the three *D. simulans* haplotypes) and one negative control. Ninety three flies were sampled in *D. melanogaster* haplotype cages, allowing two positive controls (corresponding to the two *D. melanogaster* haplotypes) and one negative control. Briefly, each digest consisted of standard squish prep with 100μl squish buffer (10 mM Tris (pH 8.2), 1 mM EDTA and 25 mM NaCl and Proteinase K added to a concentration of 200µg/ml (see Gloor *et al.* 1993). DNA extractions were conducted in 96-well plates and flies were homogenized using a pellet pestle (Kontes, Sigma, St. Louis, MO, USA) and wooden toothpick. Following digestion at 37° for 1hr, the digestion mixture was denatured by boiling at 100° for 5 min. The resulting denatured homogenate was used as the DNA template for PCR.

### Polymerase Chain Reaction (PCR)

A PCR product within the mtDNA Cytochrome Oxidase I (COI) gene was amplified using the forward primer *3593* (GAACAGTTCCCGCTTTAGGAG) and reverse primer *4528* (GCAGTTAATCGGACAGCTAATGTCCC). PCRs were conducted in 10μl volumes and reagent concentrations were as follows: 1X PCR Reaction Buffer (containing 1.5mM MgCl_2_ (Denville Scientific Inc.); 0.1mM of each dNTP (Invitrogen); 5µM of each primer (Invitrogen); 0.1U of Taq polymerase (Denville Scientific Inc.); 1μl squish prep DNA template. Thermocycling conditions were as follows: an initial incubation at 94° (2 min) followed by 30 cycles of 94° (30s), 54° (30s), 72° (45s). A final extension incubation at 72° (8 min) completed the reaction. Positive controls of haplotypes were run on each 96-well plate for the respective *Drosophila* species haplotypes.

### Restriction Fragment Length Polymorphism (RFLP) analysis

Following PCR, PCR fragments were digested to characterize haplotype using a combination of two restriction enzymes. For *D. melanogaster* haplotypes, the enzyme *AluI* (Invitrogen) was used in a restriction digest using 10μl of PCR product as reaction template. 6μl of a restriction digest mixture containing 1.5μl 10X digestion buffer (Invitrogen), 0.15μl 1X BSA, 0.2μl *AluI* restriction enzyme, and 4.15μl H_2_O was added. For *D. simulans* haplotypes, a combination of *AluI* and *RsaI* enzymes were used to characterize haplotypes. The 10μl PCR product was split into two 5μl sub-samples and each was used as template for alternative enzymes. Each 5μl PCR product was added to 3μl of a digestion mixture containing 0.75μl 10X digestion buffer (Invitrogen), 0.075μl 1X BSA, 0.1μl *AluI* or *RsaI* restriction enzyme, and 2.075μl H_2_O. All restriction digests were incubated at 37° for 5 hr. Post incubation, the independent *D. simulans* digests from the same sample were pooled for genotype (haplotype) scoring. Restricted PCR products were scored by the same researcher using agarose gel electrophoresis. Restriction patterns were confirmed with positive sample controls and *in silico* digestions of the known fragment, providing the theoretical restriction pattern. For *D. melanogaster* haplotypes, frequency estimates were based on genotypes obtained from mean = 83.73 ± 11.63 (1 SD), range= 36-93 individuals. For *D. simulans* haplotypes, frequency estimates were based on genotypes obtained from mean = 85.63 ± 8.35 (1 SD), range = 50-92 individuals.

We found the starting haplotype frequencies to vary considerably between cages, in spite of equal numbers of representatives being added at the start of the pre- and post-reperturbation experimental phases. This posed a problem for the analyses, since most population cage studies assume the starting frequency to be equal, or exactly according to the ratio of flies from distinct genetic backgrounds, even if that is not a true assumption. Our results and those of (Hutter and Rand 1995; Kilpatrick and Rand 1995) suggest this assumption is not a robust strategy. Instead, we used the estimated frequencies from the flies at the end of the starting generation as the starting frequency, since the target starting frequency was not achieved. This was partly due to flies not surviving the first generation and presumably not contributing equally to the first generation breeders.

### Statistical analysis

Statistical analyses were divided into pre- and post-perturbation phases. To estimate the selection coefficient, *s*, of each cage, we used linear models to regress the natural log of the haplotype frequency (independent variable) against generation (dependent variable) *sensu* Dykhuizen and Hartl (Dykhuizen and Hartl 1980). To determine if the overall haplotype frequency coefficient (including all four replicate cages) was significantly different from zero, we used general linear mixed-effect models on the natural log of haplotype frequency, with generation fitted as a fixed effect and cage ID fitted as a random effect. Markov Chain Monte Carlo sampling with 5000 iterations was used to calculate 95% confidence intervals (CI). Significance was judged as 95% CIs excluding zero. Log-likelihood ratio tests were performed on models with (mixed effect models: MEM) and without (linear models: LM) the random effect (Cage ID) fitted to determine if the haplotype frequency of cages was repeatable between generations within each haplotype. In other words, we asked whether cages that had relatively high haplotype frequencies in Generation 0, also had relatively high haplotype frequencies in Generations 1, 3, 5 etc., or alternatively, whether there were no repeatable effects between generations. Between generation repeatability for each cage, V_R_, was estimated as: *V_R_ = v/(v+r)*, where *v*= random effect variance across cages, and *r*= random effect residual across cages. The test statistic was 2× the difference in model Log-likelihoods (logLike_LM_-logLike_MEM_). Chi-squared tests with 1 d.f. on the test statistic gave the associated p-value. Significance was judged as *P* < 0.05.

For the analysis of the non-competitive fecundity assay, we conducted linear models to test the effects of mtDNA haplotype on offspring number. T-test statistics were used to test the difference between haplotypes compared to the model genotype, and ANOVAs were conducted to test for an overall haplotype effect. All statistical analyses and graphics were performed using the R-package software version 3.1.3 (R Core Team 2018).

### Tests of isogenicity of the nuclear backgrounds

We tested the nuclear backgrounds for any nuclear variation using diagnostic tests on RNA-seq libraries from a previous study (Mossman *et al.* 2016b; Mossman *et al.* 2017) following a method outlined in (Mossman *et al.* 2019) . We performed ‘pair’ and ‘trio’ analyses on the .bam files (samtools v.1.1.19 (Li *et al.* 2009)) in four of the ten genotypes used here (*OreR;OreR*, *si*I*;OreR*, *OreR;Aut*, *si*I*;Aut*) (Figure S1). These genotypes had been maintained in the laboratory for approximately 100 generations at the time of this experiment. We tested for: (i) transcriptome nucleotide variants that segregate between haplotypes within a nuclear background (*e.g.*, *si*I*;OreR vs. OreR;OreR*, and *si*I*;Aut vs. OreR;Aut*) (*pair* analysis), and (ii) variants that are present in the transcriptome within each of four genotypes (*trio* analysis). The pair and trio analyses provide a phred log ratio of genotype likelihoods with and without the pair or trio constraint (CLR: an integer value between 0-255). For the *pair* analysis we merged the replicate RNA-seq libraries (.bam files) and in the *trio* analysis we tested the individual replicate libraries against each other, assigning parent and offspring randomly in the trios. Inconsistent genotypes between parents and the offspring are flagged as putative variants and assigned a CLR score. We plotted the CLR of each putative variant against the linearized genome coordinates. The numerical results are described in the Supplementary Materials (Figure S2, Figure S1 and Table S1). Peaks in this nucleotide landscape indicate regions of dissimilarity between the transcriptome sequences. Higher CLR values indicate higher confidence in the difference at that transcriptome site. An extended methodology is described in the Supplementary Materials.

### Data availability

Drosophila strains are available upon request. Population cage frequency data and fecundity data are supplied as Supplementary Materials. Supplemental material available at Figshare: https://doi.org/10.25387/g3.7691939.

## Results

### Pre-perturbation

**Figure 1:**
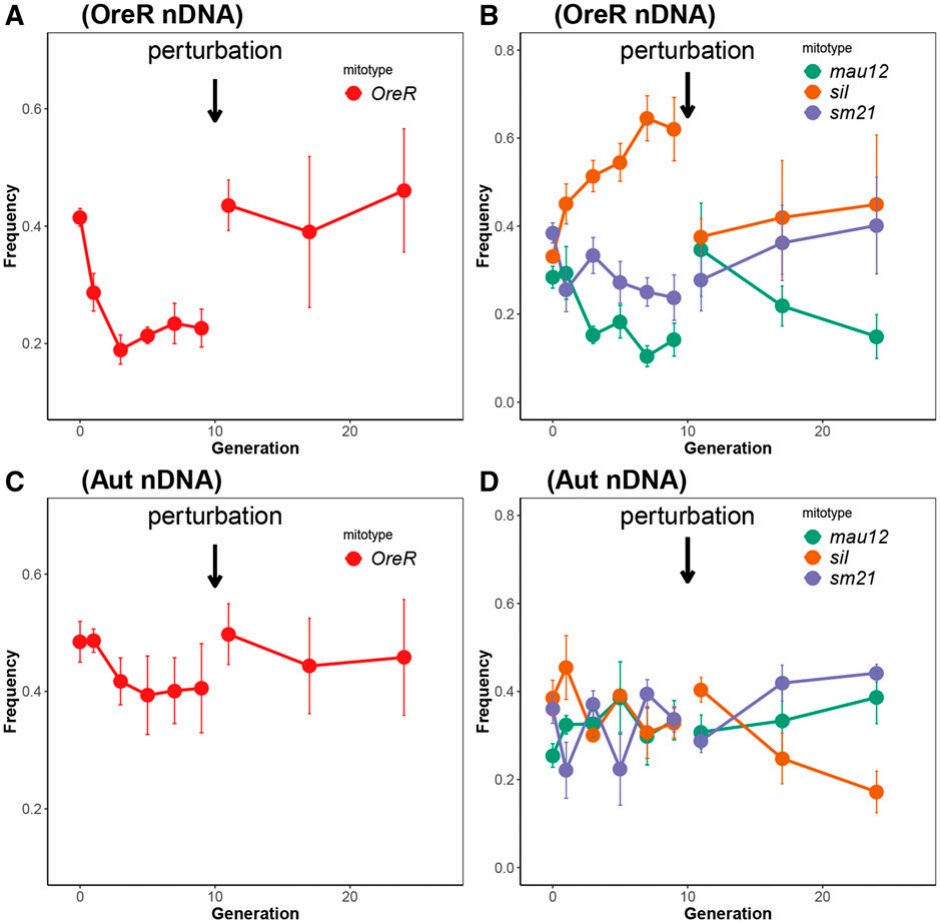
Frequencies of mtDNAs with different nDNA types as a function of generational time in population cages. (A) shows the pre- and post-perturbation frequency changes for *OreR* nDNA when the competitor mtDNA types were *OreR* and *Zm*53. Only the frequency changes of the *OreR* haplotype (red line) is shown for clarity. Figure (C) shows the pre- and post-perturbation frequency changes of *OreR* mtDNAs on the *Aut* nDNA background. (B) shows the frequencies of *D. simulans* haplotypes in the pre- and post-perturbation cages on an *OreR* nuclear background. The figure displays the frequency changes of *mau12* (green), *siI* (orange) and *sm21* (purple) haplotypes over generational time. (D) shows the frequency changes of *D. simulans* haplotypes on an *Aut* nuclear background. Selection coefficients are reported in Tables 1 and 2. Mean frequencies ± 1 SEM are shown.

For each mtDNA-type cage (*D. melanogaster* or *D. simulans*), equal numbers of individuals per haplotype were added to their allotted cages. These were 350 flies for each haplotype for the two-haplotype *D. melanogaster* cages, and 233 flies for each haplotype for the three-haplotype *D. simulans* cages. The same numbers of flies were introduced pre- and post-perturbation. However, in spite of careful counting of flies to start the population cages, there was considerable variation in the starting frequencies of mtDNA haplotypes at generation 0 (the starting generation) as determined by the PCR-RFLP assay (Figure 1). For all statistical analyses we used the haplotype frequency estimates throughout and not the absolute frequency of flies added. The former values better represent the number of flies that were alive at the end of the 1^st^ generation, since some flies did not survive the 5 days of egg laying, and would therefore not have contributed to the egg population laid in the first 5 days.

### Pre-perturbation D. melanogaster mtDNA haplotype cages

For *D. melanogaster* mtDNA haplotypes, the frequency of the *OreR* mtDNA haplotype decreased relative to the *Zim*53 mtDNA as a function of generational time with selection coefficients (mean ± 1 SE) of -0.052 (0.020) and -0.028 (0.011) for *OreR* and *Aut* nuclear types, respectively (Table 1 and Figure 1A & 1C). We report only the selection coefficients of *OreR* mtDNA because the *Zm*53 mtDNA haplotype frequency is inversely proportional to that of OreR, and is therefore redundant. The rank order (repeatability, V_R_) of mtDNA haplotype frequency differed between cages for the *OreR* nuclear type (*P* > 0.05) as a function of generation. In other words, there was significant crossing of frequency trajectories between the cages and the cage with the highest frequency at generation 0 was not necessarily the same as the cage with the highest frequency at generations 1, 3, 5, 7 and 9. In contrast, the *Aut* nuclear type cages showed a significantly repeatable rank order of cages as a function of generational time; cages with relatively high frequency at generation 0 also had relatively high frequency at generations 1,3,5,7 and 9 (*P* < 0.05, Table 1 and Figure 1C). The rank order of the mean haplotype frequency (fitness) was *Zm*53>*OreR* on both nuclear genetic backgrounds (see Figure 1A & 1C).

**Table 1:** Linear and mixed effect model results for pre- and post-perturbation selection coefficients for *D. melanogaster* mtDNA cage types. Selection coefficients for each cage (*S* cage) and the overall haplotype selection coefficient (*S* Haplotype) are shown. 95% confidence intervals are shown, along with the repeatability (V_R_) of the rank order of each cage within haplotype (see Materials and Methods). P-values (in parentheses) are from Chi-sq distributions. Bold denotes significance at α=0.05

			Pre-perturbation				Post-perturbation			
nDNA	Haplotype	Cage	*S* cage	*S* Haplotype	95% CI	V_R_	*S* cage	*S* Haplotype	95% CI	V_R_
***OreR***	***OreR***	1	−0.102 (0.051)	**−0.052 (0.020)**	**−0.092 to −0.012**	<0.001	−0.046 (0.062)	−0.001 (0.019)	−0.054 to 0.054	**0.63**
		2	−0.026 (0.030)			(*P* = 0.91)	0.007 (0.006)			(*P* = 0.049)
		3	−0.054 (0.028)				0.010 (0.056)			
		4	−0.028 (0.046)				0.024 (0.010)			
***Aut***	***OreR***	1	−0.047 (0.030)	**−0.028 (0.011)**	**−0.055 to −0.003**	**0.55**	−0.027 (0.007)	−0.010 (0.011)	−0.042 to 0.022	0.62
		2	0.025 (0.010)			(*P* = 0.003)	−0.042 (0.012)			(*P* = 0.053)
		3	−0.019 (0.006)				0.013 (0.006)			
		4	−0.071 (0.012)				0.015 (0.028)			

### Post-reperturbation D. melanogaster haplotype cages

In contrast to the pre-perturbation cages, there were no significant relationships between haplotype frequency and generational time for *D. melanogaster* mtDNA haplotypes in either *OreR* or *Aut* nuclear backgrounds (*P* > 0.05 in all cases). In the *OreR* nuclear genetic background, the rank order of *OreR* mtDNA frequency was significantly repeatable over time in the pre-perturbation cages. In the post-perturbation cages, however, there was no consistent directionality of selection and therefore no significant pattern that would suggest consistent selection on mtDNA haplotype. The *OreR* mtDNA haplotype frequency in the *Aut* nuclear background showed no repeatable rank order over time between cages (Table 1). However, the post-reperturbation cages demonstrated a consistent rank order of mean haplotype frequency, similar to the pre-perturbation cages. In both nuclear genetic backgrounds, *Zm*53 showed greater mean fitness than *OreR* (*Zm*53>*OreR*) (see Figure 1A & 1C).

### Pre-perturbation D. simulans mtDNA haplotype cages

For *D. simulans* mtDNA haplotypes, there were clear differences between the selection coefficients of mtDNA haplotypes on alternative nuclear genetic backgrounds. For *OreR* nuclear genetic background, selection coefficients for *mau*12 (s= -0.106 ± 0.025) and *sm*21 (s = -0.044 ± 0.02) mtDNAs were negative and significant, whereas the *si*I (*s* = 0.062 ± 0.01) mtDNA significantly increased in frequency (Figure 1B and Table 2). All three selection coefficients were significantly different from zero at *P* < 0.05. The rank orders of haplotype frequency for all except one of the cage sets were non-repeatable, indicating that there was significant crossing over between the norms of reaction of cages within each haplotype between generations (Table 2). For the *OreR* nuclear type, the rank order of the mean haplotype frequencies at the end of the pre-perturbation episode was *si*I>*sm*21>*mau*12 (see Figure 1B).

**Table 2:** Linear and mixed effect model results for pre- and post-perturbation selection coefficients for *D. simulans* mtDNA cage types. Selection coefficients for each cage (*S* cage) and the overall haplotype selection coefficient (*S* Haplotype) are shown. 95% confidence intervals are shown, along with the repeatability (V_R_) of the rank order of each cage within haplotype (see Materials and Methods). P-values (in parentheses) are from Chi-sq distributions. Bold denotes significance at α=0.05

			Pre perturbation				Post perturbation			
nDNA	Haplotype	Cage	*S* cage	*S* Haplotype	95% CI	V_R_	*S* cage	*S* Haplotype	95% CI	V_R_
***OreR***	***mau*12(*si*III)**	1	−0.161 (0.057)	**−0.106 (0.025)**	**−0.156 to −0.056**	<0.001	0.037 (0.030)	−0.085 (0.040)	−0.185 to 0.009	0.37
		2	−0.155 (0.016)			(*P* = 1.0)	−0.242 (0.050)			(*P* = 0.28)
		3	−0.024 (0.022)				−0.096 (0.007)			
		4	−0.085 (0.075)				−0.039 (0.016)			
	***si*I (*si*I)**	1	0.058 (0.019)	**0.062 (0.010)**	**0.042 to 0.084**	0.29	−0.003 (0.001)	−0.000 (0.018)	−0.055 to 0.051	**0.64**
		2	0.079 (0.022)			(*P* = 0.11)	0.063 (0.027)			(*P* = 0.04)
		3	0.037 (0.018)				−0.004 (0.033)			
		4	0.074 (0.021)				−0.057 (0.002)			
	***sm*21(*si*II)**	1	0.030 (0.046)	**−0.044 (0.020)**	**−0.085 to −0.0004**	0.28	−0.008 (0.009)	0.025 (0.037)	−0.053 to 0.105	<0.001
		2	−0.109 (0.035)			(*P* = 0.11)	−0.089 (0.044)			(*P* = 0.85)
		3	−0.031 (0.014)				0.136 (0.077)			
		4	−0.068 (0.034)				0.062 (0.022)			
***Aut***	***mau*12(*si*III)**	1	0.017 (0.034)	0.013 (0.019)	−0.026 to 0.052	<0.001	0.023 (0.010)	0.017 (0.019)	−0.033 to 0.066	0.39
		2	0.043 (0.052)			(*P* = 1.0)	0.006 (0.084)			(*P* = 0.25)
		3	0.041 (0.034)				0.001 (0.026)			
		4	−0.050 (0.029)				0.039 (0.043)			
	***si*I (*si*I)**	1	−0.037 (0.030)	−0.029 (0.018)	−0.064 to 0.009	<0.001	−0.179 (0.013)	**−0.083 (0.024)**	**−0.143 to −0.018**	0.48
		2	−0.024 (0.030)			(*P* = 0.72)	−0.048 (0.025)			(*P* = 0.15)
		3	−0.074 (0.051)				−0.049 (0.014)			
		4	0.019 (0.014)				−0.055 (0.048)			
	***sm*21(*si*II)**	1	0.064 (0.118)	0.022 (0.034)	−0.051 to 0.090	0.10	0.037 (0.006)	**0.033 (0.010)**	**0.013 to 0.054**	<0.001
		2	−0.020 (0.070)			(*P* = 0.55)	0.031 (0.021)			(*P* = 0.84)
		3	0.031 (0.046)				0.050 (0.037)			
		4	0.014 (0.025)				0.015 (0.004)			

For the *Aut* nuclear genetic background, there were no significant relationships between haplotype frequency and generational time across all *D. simulans* mtDNA haplotypes (*P* > 0.05 in all cases, Table 2 and Figure 1D). For the *Aut* nuclear type, the rank order of the mean haplotype frequencies at the end of the pre-perturbation episode was *si*I=*sm*21=*mau*12 (all three haplotypes had a mean frequency of 33%, see Figure 1D).

### Post-reperturbation D. simulans haplotype cages

For *D. sim*ulans mtDNA haplotypes, the post-reperturbation cages showed clear differences between the *OreR* nuclear background and the *Aut* nuclear background. In the *OreR* nuclear background, there was no evidence of selection on any haplotype, since all the haplotypes demonstrated non-significant selection coefficients (*P* > 0.05, Table 2). For the *OreR* nuclear type, the rank order of the mean haplotype frequencies at the end of the post-reperturbation episode was *si*I>*sm*21>*mau*12 (see Figure 1C), the same as in the pre-perturbation experimental episode.

In the *Aut* nuclear background, there were significant relationships between the frequency of *si*I (s= -0.083 ± 0.024) and *sm*21 (s= 0.033 ± 0.010) haplotypes and generational time, however, the selection coefficient of the *mau*12 mtDNA haplotype did not significantly differ from zero (Table 2 and Figures 1D). For the *Aut* nuclear type, the rank order of the mean haplotype frequencies at the end of the post-reperturbation episode was *sm*21>*mau*12>*si*I (see Figure 1D).

### Non-competitive fecundity effects

**Figure 2:**
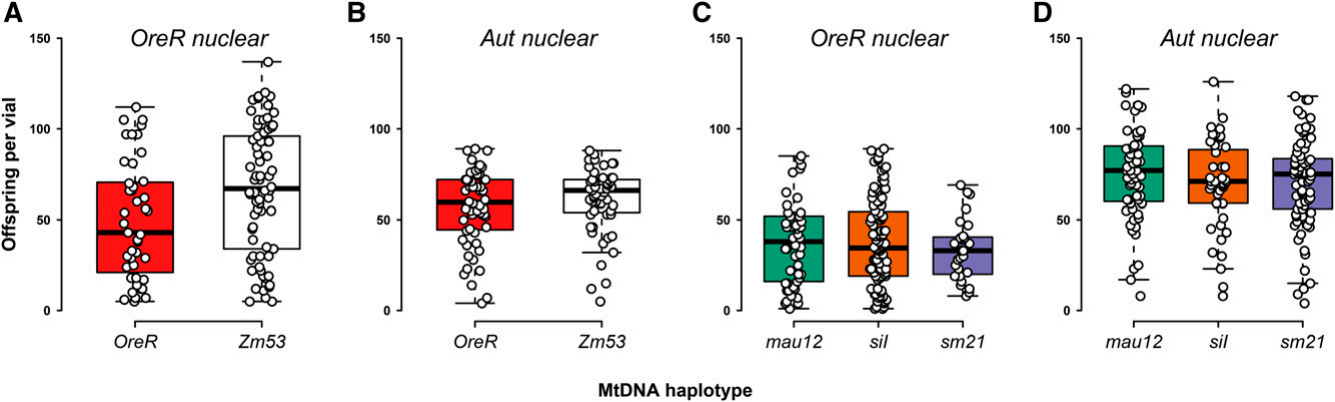
Mito-nuclear introgression effects on isofemale offspring production in a non-competitive five-day egg-laying period. Panels (A) and (B) show *D. melanogaster* mtDNAs on *OreR* and *Aut* nDNAs, respectively. Panels (C) and (D) show isofemale offspring numbers for *D. simulans* mtDNAs on *OreR* and *Aut* nDNAs, respectively. For comparisons within nDNA types, *D. simulans* mtDNA isofemales produced significantly more offspring than the *D. melanogaster* mtDNA types on the *Aut* background (comparison between (B) and (D); Table 4). *D. melanogaster* mtDNA-bearing isofemales produced significantly more offspring than the *D. simulans* mtDNA-bearing isofemales in the *OreR* nDNA background (comparison between (A) and (C); Table 4). Within a cage type, the only significant difference in offspring numbers were between *Zm*53 and *OreR* haplotypes on *OreR* nDNA (Figure 2A and Table 3).

For the analyses of offspring number and haplotype variation for cages of known nuclear type, we found large differences between cage types for fecundity in the generation prior to reperturbation (Tables 3 and 4, Figure 2). In the *OreR* nuclear background cages, there was a significant difference between the number of offspring between *D. melanogaster*-type and *D. simulans*-type mtDNAs. *OreR* nDNA isofemales with *D. melanogaster* mtDNAs produced significantly greater offspring than isofemales harboring *D. simulans* mtDNAs (Table 4). In contrast, isofemales with the *Aut* nuclear background produced statistically greater numbers of offspring when harboring *D. simulans*-type mtDNAs (Table 4), and greater numbers than the *OreR* nuclear background overall. Within the cage types, the results were less dramatic (Table 3 and Figure 2). In *OreR* nDNA cages there were no statistical differences between the numbers of offspring per isofemale with different *D. simulans* mtDNA haplotypes (Table 3 and Figure 2C). There was also no effect of mtDNA haplotype on offspring number in the *Aut* nDNA background with *D. simulans* mtDNAs (Table 3 and Figure 2D). For the analyses of *D. melanogaster* type mtDNAs and offspring number, there were clear contrasts between the nuclear backgrounds. In the *OreR* nDNA background, the *Zm*53 mtDNA-bearing isofemales produced statistically greater numbers of offspring than the *OreR* mtDNA-bearing isofemales (Table 3 and Figure 2A). There was no effect of mtDNA haplotype on offspring number when the mtDNAs were on *Aut* nDNA genetic backgrounds (Table 3 and Figure 2B).

**Table 3:** MtDNA haplotype effects on isofemale offspring numbers for the four experimental cage types. We report results from models of individual haplotype performance against the model haplotype (*mau*12 in *D. simulans* types and *OreR* in *D. melanogaster* types) and conducted T-tests. Coefficients (β ± SE), T-test statistics and P-values are shown. We also report the effects of haplotype in an ANOVA analysis, where degrees-of-freedom, sum-of-squares, R^2^, F statistics and P-values are shown. Bold denotes significance at α=0.05

		T-test (Individual haplotypes against model)				ANOVA (Haplotype effect)					
Experimental mtDNA types	Nuclear type (intercept)	mtDNA type	β (± SE)	T	P	Model term	d.f.	Sum-of-squares	R^2^	F	P
*D. simulans*	*OreR*	*si*I	0.247 (3.99)	0.062	0.95	mtDNA	2	355	0.004	0.339	0.71
	37.18 (*mau*12)	*sm*21	−3.716 (5.40)	−0.69	0.49	Residuals	171	89384			
											
	*Aut*	*si*I	−4.901 (4.97)	−0.99	0.33	mtDNA	2	776	0.007	0.624	0.54
	74.42 (*mau*12)	*sm*21	−4.077 (4.34)	−0.94	0.35	Residuals	175	108762			
											
*D. melanogaster*	*OreR*	*Zm*53	**17.113 (6.651)**	**2.57**	**0.01**	mtDNA	1	7801	**0.057**	**6.620**	**0.01**
	49.56 (*OreR*)					Residuals	111	130810			
											
	*Aut*	*Zm*53	4.303 (3.719)	1.16	0.25	mtDNA	1	514	0.012	1.339	0.25
	56.68 (*OreR*)					Residuals	109	41841			

**Table 4:** Linear model results for isofemale number of offspring eclosed against mtDNA species type. Results for *OreR* and *Aut* nuclear types are shown. The model coefficients (β ± SE) are compared against the respective values for *D. melanogaster* mtDNAs (intercept= 60.16 for *OreR* nuclear type; intercept= 58.81 for *Aut* nuclear type). In the *OreR* nuclear background, isofemales with *D. simulans* mtDNAs produced significantly fewer offspring than isofemales with *D. melanogaster* mtDNAs. In the *Aut* nuclear background, isofemales with *D. simulans* mtDNAs produced significantly more offspring than isofemales with *D. melanogaster* mtDNAs (see Figure 3). In both nuclear backgrounds, there was a significant effect of mtDNA species type (*D. simulans vs. D. melanogaster*) on isofemale offspring numbers. Degrees-of-freedom, R^2^, F-statistics and P-values are shown. Bold denotes significance at α=0.05

	T-test (Individual haplotypes against model)				ANOVA (Haplotype effect)					
Nuclear type	Term against model	β (± SE)	T	Nuclear type	Model term	d.f.	Sum-of-squares	R^2^	F	P
*OreR*	*D. simulans* mtDNA	**−23.44 (3.42)**	**−6.85**	*OreR*	mtDNA species	1	37644	**0.145**	**46.98**	**4.43e-11**
					Residual	285	228350			
										
*Aut*	*D. simulans* mtDNA	**12.68 (2.78)**	**4.56**	*Aut*	mtDNA species	1	10998	**0.067**	**20.78**	**7.63e-6**

### Nuclear genome variation is present in the genetic stocks

**Figure 3:**
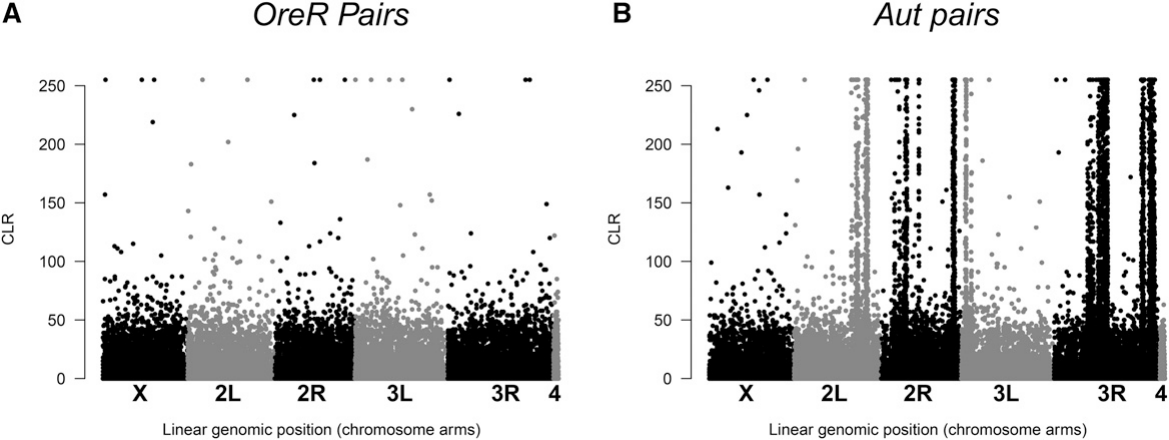
Whole transcriptome SNP variation between haplotypes within nuclear genetic backgrounds (pairs analysis). (A) shows the relative chromosome position of putative transcriptome-wide SNPs on the abscissa against the CLR (confidence) score (see main text) on the ordinal axis for the between-*OreR* comparison (*si*I*;OreR vs. OreR;OreR*). (B) shows the between-*Aut* nuclear haplotype comparison (*si*I*;Aut vs. OreR;Aut*).

**Figure 4:**
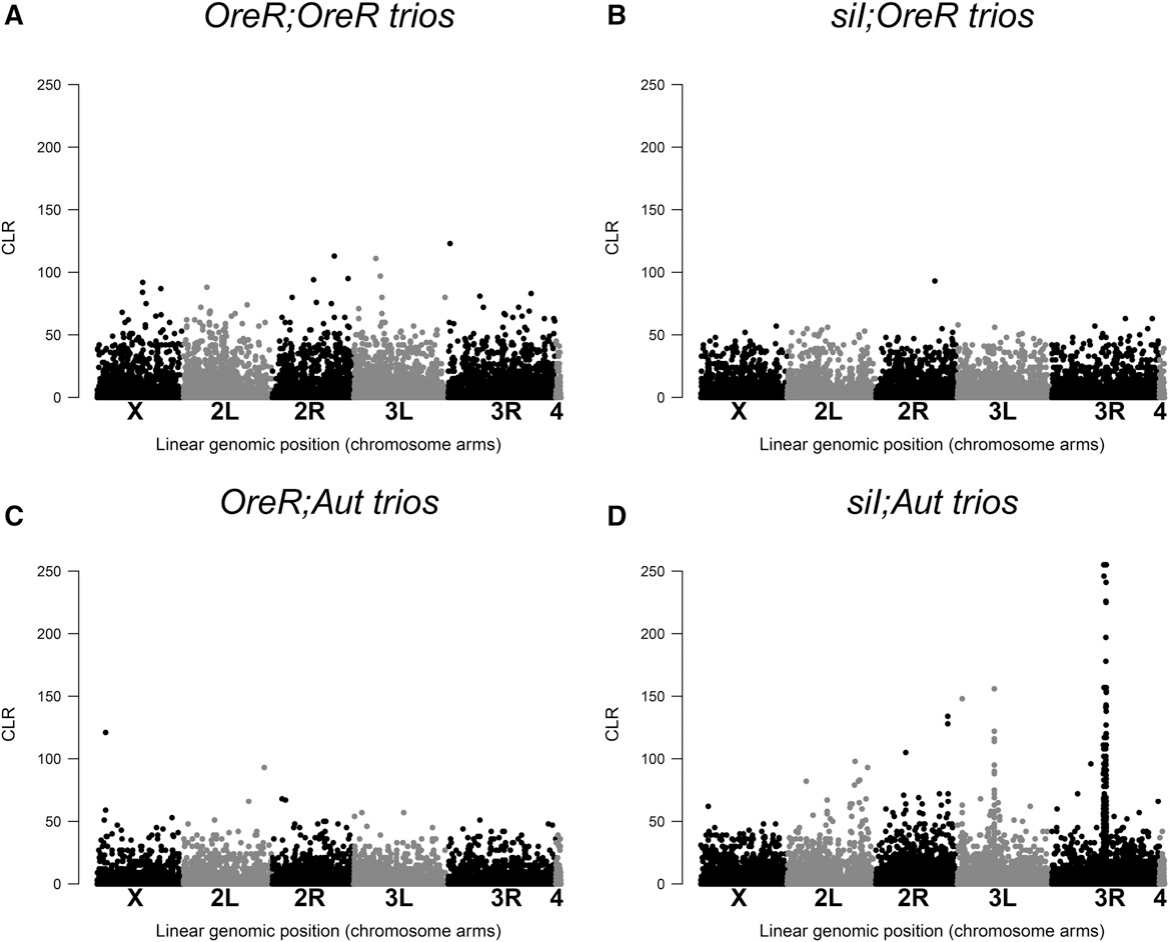
Whole transcriptome SNP variation within mitonuclear genotypes (trios analysis). The trio analyses results are shown for *OrR;OreR* (A), *si*I*;OreR* (B), *OreR;Aut* (C), and *si*I*;Aut* (D). See Supplementary Materials for numerical estimates across CLR values (Figures S2, S3 and Table S1). The *si*I*;Aut* genotype shows evidence of some SNP variation, particularly in the 3R chromosome arm.

Our pairs and trios analyses suggest there are low levels of nucleotide variation within and between *si*I*;OreR* and *OreR;OreR* (Figures 3 and 4; Table S1). There are regions of the *Aut* nuclear background that contain clustered variants between the *si*I*;Aut* and *OreR;Aut* haplotypes (Figure 3B). On closer inspection in trio analyses, the between-haplotype variation identified in the pairs analysis is likely caused by SNPs that are segregating solely in the *si*I*;Aut* genotype libraries (Figure 4D; Table S1).

## Discussion

The pre- and post-perturbation selection coefficient results suggest that population cages showed several alternative modes of changing mtDNA haplotype frequencies as a function of generational time. For the *D. melanogaster* mtDNA haplotypes, both of the pre-perturbation cage treatments showed frequency changes consistent with a selection regime. However, in the post-perturbation phase of the experiment, these cages no longer showed consistent and unidirectional mtDNA haplotype frequency changes with time, suggesting mtDNA frequencies were changing by a more neutral process such as genetic drift. In contrast, the *D. simulans* cages showed an alternative profile, that was also nuclear genetic background-specific. In the *OreR* nDNA background, the pre-perturbation cages showed evidence of unidirectional change, and the selection coefficients for each haplotype were significantly different from zero. Following reperturbation, these cages no longer showed a unidirectional and significant selection coefficient, consistent with a neutral process. In the *Aut* nuclear background, the pre-perturbation populations showed evidence consistent with neutral processes, whereas post-perturbation, two of the three cage types showed consistent and unidirectional selection coefficients. We found female fecundity differences between haplotypes on alternative nuclear backgrounds could only partly explain haplotype frequency changes in the population cages. We discuss these results in the context of female fecundity, the differences pre- and post-reperturbation, and the implications of G x G and G x G x E interactions affecting fitness in population cages. We finally discuss whether there was evidence to support our initial predictions based on the first principles of phylogenetic relatedness of haplotypes and its possible phenotypic consequences.

### Female fecundity - a trait driving frequency changes in population cages?

There was considerable variation in the number of offspring produced in the non-competitive cage fecundity assays. The results suggest two main effects of nuclear and mtDNA co-variation. First, with *OreR* nuclear backgrounds, the greatest numbers of offspring were produced with the same species mtDNAs. That is, isofemales with *OreR* nDNA and *D. melanogaster* mtDNAs were statistically more fecund than isofemales with *D. simulans* mtDNAs, consistent with our co-adapted prediction. The opposite effect was evident with *Aut* nuclear genetic backgrounds; *D. simulans* mtDNA haplotypes produced significantly greater numbers of offspring than individuals with the co-evolved *D. melanogaster* mtDNAs, suggesting the nuclear variant modified the mtDNA fecundity results in an epistatic manner. We were surprised that the effects of *D. simulans* mtDNAs were advantageous in a *D. melanogaster* nuclear background, in spite of originating in a separate species and contrary to our prediction. However, we have previously recognized this effect in some of these genotypes for egg production (Meiklejohn *et al.* 2013). Comparisons between nuclear backgrounds in Figures 2A and 2B suggest that *OreR* mtDNA haplotypes perform more variably on *OreR* nDNA, because in the *Aut* nuclear background there is no significant difference between haplotypes (Table 3). Overall, we found that the population cage that demonstrated the greatest deviation from haplotype equality at an early stage of the pre-perturbation experiment was the *OreR* nuclear background with *D. melanogaster* mtDNA haplotypes. We found a concurrent significant difference between the performance of the *D. melanogaster* mtDNA haplotypes in offspring numbers, suggesting that only in cages where there are clear and statistical differences in fecundity between the competing genotypes, do we see clear signatures of selection, which is perhaps predictable.

### Inconsistent cage behavior pre-and post-perturbation

Compared to the Ballard and James (2004) study, which found the fitness differences between haplotypes in pre-perturbation phase to be extensive and repeatable (in *D. simulans* nDNA backgrounds), we did not find the same haplotype effects when the *D. simulans* haplotypes are resident on *D. melanogaster* nDNA genetic backgrounds. In the Ballard and James study (2004), the *si*II haplotype showed the strongest increase in frequency, both pre- and post-perturbation, whereas in the present study, there was no significant increase in *si*II haplotype frequency on the *Aut D. melanogaster* nuclear background. In fact, on an *OreR D. melanogaster* nuclear background, the *si*II haplotype significantly decreased over time pre-perturbation, demonstrating the opposite effect and therefore suggesting a mtDNA-nDNA epistasis for fitness that is mediated by the *D. melanogaster* nuclear variant. Although we selected the same general haplotypes to test for repeatable fitness effects in different nuclear genetic backgrounds, there are obviously ‘species’ genetic variants between *D. simulans* and *D. melanogaster* nuclear backgrounds that confer fitness effects when in their native (co-evolved) *D. simulans* mtDNA-nDNA configuration. We further identified SNP variation within the *si*I*;Aut* genotype. These genetic differences could partly explain why we did not observe the same rank order of fitness of *D. simulans* haplotypes in both of the *D. melanogaster* nuclear backgrounds tested here. Furthermore, sporadic mtDNA-nDNA epistases may have been generated between generations at variable transcriptome sites and these unpredictable events are consistent with no overall signature of selection.

Why are there differences between mtDNA haplotype competitiveness depending on the nuclear (species) background? While we used balancer chromosomes to precisely place isogenic nuclear chromosomes on the mtDNAs (see Montooth *et al.* 2010), we still observed an effect of erosion of potentially beneficial (linked) markers between the *OreR* nuclear background and *Zm*53 *D. melanogaster* mtDNAs with generational time (see Figure 1A). In the same cage type (*OreR* nuclear) the *Zm*53 mtDNA frequency increased rapidly in the preliminary generations pre-perturbation, but formed an asymptote around generation four, an effect similar to other population cage frequency changes (Kilpatrick and Rand 1995). For the following generations, there was no apparent change in frequency and the haplotype did not fix in any of the four replicate populations. The results from the present population cage study are supported by the differences we observed between mito-nuclear types for offspring numbers (see above). In another population cage experiment (Kilpatrick and Rand 1995) the effect of decelerating haplotype frequency change could be explained by the decay of spurious linkage disequilibrium (LD: mtDNA-nDNA association) during the early generations of the experiment. Essentially, the ‘conditional hitchhiking’ in Kilpatrick and Rand (1995) arose through initial association between hybrid nDNA, and mtDNA variants. In theory each generation post- hybridization saw an erosion of LD by a factor of *r* = 0.5 on average, which was evident in the data, hinting that a nuclear genetic component was contributing to the main haplotype frequency change effect. In the same study any residual LD between mtDNA and nDNA, which was present in the pre-perturbation phase, was not sufficient to cause haplotype frequencies to significantly change post-repurturbation. In the present study we aimed to use balancer chromosome introgression to minimize this occurrence but we did identify some genetic variation within mitonuclear genotypes and between haplotypes within a nuclear background (*Aut*) that had persisted or had been generated since the genotype construction ∼100 generations prior to the transcriptome analyses. Despite careful genotype construction, the deleterious mutation rate of *U*∼1-1.2 per diploid genome per generation (Haag-Liautard *et al.* 2007; Keightley *et al.* 2009; Charlesworth *et al.* 2004) all but ensures that any experimental genetic study will have fitness-related LD that may decay over time.

### Relaxed selection in the experimental procedure?

Interestingly, in *D. simulans* nuclear backgrounds, *si*I females have a significantly shorter egg-to-puparium development time than *si*II or *si*III females (James and Ballard 2003), which would presumably confer a competitive advantage in a population cage. However, *si*I haplotype frequencies are dramatically reduced as a function of generational time in population cages (Ballard and James 2004), suggesting that development time is not a trait closely coupled to competitive advantage in population cages and is more likely linked to a lower probability of survival in this haplotype (James and Ballard 2003).

A more clear effect of female fecundity driving frequency change was observed by Hutter and Rand (1995), where a population cage experiment of mito-nuclear introgressed *D. pseudoobscura* and *D. persimilis* revealed egg production rate differences between genotypes could explain the competitive exclusion of the non-co-evolved *D. persimilis* mtDNA when it was introgressed with *D. pseudoobscura* nuclear DNA; the co-evolved *D. pseudoobscura* mtDNA-nDNA flies had a significant fertility advantage. The discrepancy between haplotype ‘competitiveness’ and female fertility measures in James and Ballard (2003) and Hutter and Rand’s (1995) studies suggest large fitness differences may be required to demonstrate significant changes in haplotype frequencies. Alternatively, non-competitive fitness assays may poorly predict the competitiveness of haplotypes in the population cage environment. The present study also found statistical differences in female fecundity, although we could not explain the differences in haplotype frequency changes by female fecundity alone.

We favor the hypothesis that selection on female life history traits has been relaxed in the current study. This has potentially allowed low fitness genotypes to co-exist with high fitness genotypes in cages when there is no significant fecundity difference between mtDNA haplotypes (as observed for all cages except *OreR* nuclear DNA with *D. melanogaster* mtDNAs). For example, if there was a significantly greater developmental time in one haplotype - as evidenced in the w501 *D. simulans* mtDNA in Montooth *et al.* (2010) and Meiklejohn *et al.* (2013)- that haplotype would shift to low frequency rapidly if the experimental transition between generations was before eclosion or reproductive maturation in that female’s mtDNA haplotype. On the other hand, allowing females to lay eggs for five days instead of three or four, we may have effectively relaxed the selection for early development and fecundity, and therefore potentially greater (and possibly equal) offspring numbers could be produced across all female haplotypes, providing no consistent evidence of a selection process. We kept the generation time constant in an effort to minimize any stochastic variation in egg laying between generations that could introduce density-induced effects on fitness (Clark and Feldman 1981). However, it is possible that the *Aut* nDNA flies, which have generally higher fecundity, laid more eggs in the 5 day laying period. We found that the population sizes in cages did fluctuate over time and this may have resulted in variable larval densities and thus variable larval competition, possibly mitigating any haplotype advantage of faster development.

### G X G

The earliest investigations of mtDNA selection suggested that mtDNA behaves as a selectively neutral genetic marker (reviewed in (Rand 2001)) and (Ballard and Whitlock 2004)). Recent elegant studies have challenged this dogma and shown quite clearly strong non-neutral components of mtDNA selection (*e.g.*, Ballard and James 2004). The present study adds further evidence for the complexity of mtDNA evolutionary dynamics and suggests that any selection advantage for fitness of a given mtDNA haplotype is dependent on the nDNA variant it is inherited with (in the case of ostensibly isogenic nDNA). Furthermore, we suggest any mtDNA haplotype selection is also dependent upon residual genetic variation *within* a population of nDNAs, which can be uncoupled through generational time via the erosion of LD between beneficial or deleterious mtDNA and nDNA variants (*sensu* conditional hitchhiking: (Kilpatrick and Rand 1995)). For example, the same *D. simulans* haplotypes used in Ballard and James (2004), when present on alternative *D. melanogaster* nDNAs, do not demonstrate repeatable selection either between nDNA types or pre- and post-repurturbation. In fact, we found that the rank order of the mean haplotype fitness was variable both between nDNA types and between pre- and post-perturbation experimental phases. This suggests that mtDNA x nDNA interactions are important to reveal or conceal fitness advantages associated with mtDNA selection, and that repurturbation can modify previously evident haplotype selection or neutrality.

### G X G X E

The present study determined gross measures of fitness between mtDNA haplotypes using the change in haplotype frequency as a proxy of ‘fitness’. It is possible that different population cages (*e.g.*, *OreR* nDNA *vs. Aut* nDNA) can modify their environment in different ways thus providing alternative environmentally-based selection landscapes (Dobzhansky and Spassky 1944). We found some evidence for this possibility in the *Aut* nDNA background, in which females are statistically more fecund than *OreR* nDNA females, probably altering the larval developmental environment. In the *Aut* nDNA background, *D. simulans* haplotypes demonstrated clear differences in selection coefficients pre- and post reperturbation. In the pre-perturbation phase, haplotypes showed dramatic stochastic fluctuating changes in frequency between generations whereas the post-perturbation phase showed more directional changes consistent with selection (Table 2 and Figure 1C). This may have arisen through nDNA variation being present and maintained pre-perturbation, essentially reducing the selection on mtDNA haplotype. One way nDNA variation may have been maintained is via a stochastic larval environment between generations. Alternatively, any nDNA variation (which was likely present in our cages) at mtDNA-interacting loci could have overridden the main mtDNA haplotype effect. Following reperturbation, residual nDNA variation, if greatly reduced through a genetic bottleneck, may have sensitized the mtDNA-nDNA gene complex to selection. In contrast, the *OreR* nDNA cages showed directional haplotype frequency change pre- perturbation, then no directional change post- perturbation. This may again be explained by gene x gene x environment interactions (G x G x E) (Arnqvist *et al.* 2010), which are known to be pervasive modifiers of fitness (Mossman *et al.* 2016a). We aimed to minimize environmental variation by maintaining a constant population size throughout and a consistent environment for egg laying, although the population sizes were evidently variable across generations. Therefore, larval density may have been considerably variable between nDNA types with a resulting environmental interaction modifying any main effect of mtDNA or nDNA-mtDNA epistasis for fitness. We were not able to assess the egg to larval to puparium to adult survival parameters in this investigation to test this hypothesis.

### Any evidence to support phylogenetic predictions?

We formulated three basic predictions about expected fitness in the mtDNA-nDNA introgressed flies. These were: (i) there would be greater divergence in phenotypes in the *D. simulans* clade than in the *D. melanogaster* clade; (ii) there would be more phenotypic divergence between clades than within a clade; and (iii) the non-coevolved mtDNA haplotypes (in the *D. simulans* clade) will demonstrate deleterious phenotypes if compared to *D. melanogaster* mtDNA haplotypes.

Overall, we found some support for these prediction, although due to the inconsistent behavior of cages in pre- and post-reperturbation episodes of the experiment, we have to conclude that fitness as measured in population cages is a somewhat labile trait. Likewise, the female fecundity assays showed support for the predictions in some cases and contrary evidence in other cases. For female fecundity our first prediction is largely unsupported. We found the only significant difference in fecundity within a cage type to be between *D. melanogaster* haplotypes, and not the predicted *D. simulans*, with its greater level of mtDNA genetic polymorphism. For our second prediction we found some evidence of greater offspring number divergence between clades than within clades; in both nuclear types there were significant differences in offspring numbers that was associated with mtDNA species, whereas only one of the four cages demonstrated within-species differences. Our third prediction is supported in the *OreR* nuclear background as *D. melanogaster* mtDNA haplotypes performed better than *D. simulans* haplotypes. However, the *Aut* nuclear background reversed this effect, and *D. simulans* haplotypes outperformed the *D. melanogaster* (co-evolved) counterparts, contrary to our prediction. Generally, the largest effect on offspring numbers across all treatments was the variation associated with alternative nuclear background on *D. simulans* haplotypes.

In conclusion, there were no consistent patterns of frequency change over generational time for introgressed mito-nuclear genotypes. We did not observe patterns similar to those in previous studies which used the same haplotypes (*D. simulans*) but on alternative (conspecific *D. simulans*) nuclear backgrounds. We did find that the one cage that showed a strong and significant change in haplotype frequency was the same cage that possessed haplotypes with significantly different female fecundity in a non-competitive context. We suggest this fecundity difference has large implications for the strength of frequency change, and that competing haplotypes with very similar female fecundity will likely result in non-repeatable selection coefficients. Broadly speaking, genotype effects are likely to be sensitive to both interacting genes and the environments which they are in dialogue with and as a main consequence genetic variants may not display the same fitness effects under all experimental conditions. Furthermore, while mtDNA polymorphisms confer fitness effects in some nuclear backgrounds, they may display neutral effects when paired with alternative nDNAs.
